# 3D Printing of Polyamide to Fabricate a Non-Metal Clasp Removable Partial Denture via Fused Filament Fabrication: A Case Report

**DOI:** 10.3390/ijerph18168241

**Published:** 2021-08-04

**Authors:** Sebastian Spintzyk, Roman Schmunk, Pablo Kraemer Fernandez, Fabian Huettig, Alexey Unkovskiy

**Affiliations:** 1 Section Medical Materials Science and Technology, Tuebingen University Hospital, 72076 Tübingen, Germany; Sebastian.spintzyk@med.uni-tuebingen.de; 2 Department of Prosthodontics at the Centre of Dentistry, Oral Medicine and Maxillofacial Surgery with Dental School, Tuebingen University Hospital, 72076 Tübingen, Germany; Roman.schmunk@med.uni-tuebingen.de (R.S.); pablo.kraemer-fernandez@med.uni-tuebingen.de (P.K.F.); fabian.huettig@med.uni-tuebingen.de (F.H.); 3 Department of Prosthodontics, Geriatric Dentistry and Craniomandibular Disorders, Charité-Universitätsmedizin Berlin, Corporate Member of Freie Universität and Humboldt-Universität zu Berlin, 10117 Berlin, Germany; 4 Department of Dental Surgery, Sechenov First Moscow State Medical University, 119991 Moscow, Russia

**Keywords:** polyamide, PA-12, valplast, removable partial denture, additive manufacturing

## Abstract

The fabrication of a non-metal clasp removable partial denture (RPD) using polymethylmethacrylate in a fully digital workflow has been reported. According to some studies, the polyamide material may be alternatively used for this purpose. The authors are unaware of any reports concerning the additive manufacturing of polyamide. The current proof-of-concept dental technique describes the pathway to construct the non-metal clasp RPD using intraoral scanning and fused filament fabrication (FFF) printing of gingiva-colored polyamide. The present RPD showed acceptable fit and sufficient retention and was considered a valid temporary treatment option.

## 1. Introduction

Despite advancements in oral implantology, the utilization of removable partial dentures (RPDs) for rehabilitation of partially edentulous patients remains a valid treatment option [1,2]. In order to achieve a better esthetical outcome, the utilization of non-metal clasps has been approved for retention of RPDs and reported in a row of studies [3,4]. In 1950, the polyamide-12 (PA-12, also known as “nylon”) material was introduced to the dental market. Some recent studies have investigated the polyamide material as an alternative to polymethylmethacrylate (PMMA) and reported the nylon material to have good thermo-mechanical properties but insufficient elastic modulus [5,6]. It has been claimed that nylon, due to its crystalline structure, is less soluble in solvents, has high heat resistance, and is toxicologically safe for patients with a resin monomer allergy [7,8]. Regarding retention capacities, the nylon clasps may provide sufficient retention compared to metal clasps. However, they necessitate a greater undercut and specific design [9].

In the last decade, the dental field has been significantly impacted by computer-aided design and manufacturing (CAD/CAM), and fabrication of partial and complete dentures utilizing intraoral scanning as a source of clinical data has been reported [10,11]. The feasibility of non-clasp RPDs fabrication in a fully digital workflow has also been proven using PMMA material [12,13].

The fused filament fabrication (FFF) method, also widely known as fused deposition modeling (FDM), has been widely applied in the medical field for polymer printing, as well as for dentures manufacturing [14,15,16,17]. FDM printing of ceramic-filled polyamide has also been attempted [18]. A pilot clinical case demonstrated the 3D printing of a nylon prosthesis in white color in a semi-analog way using a plaster cast [19]. However, the current literature lacks any reports on nylon prostheses fabrication using CAD/CAM in a completely digital workflow.

The present clinical case demonstrates a proof-of-concept production of a small RPD in the molar region as a temporary solution from gingiva-colored polyamide material using intraoral scanning and FFF.

## 2. Case Report

A female patient was referred to the Department of Prosthodontics at the XXXX to receive an implant-supported crown. Due to unavoidable circumstances, she could not start the treatment at the same time. In order to prevent the elongation and any movements of neighboring teeth, a polyamide RPD was proposed as an interim solution. After information about potential solutions and current developments, she gave her informed written consent to wear the interim RPD.

**Figure 1:**
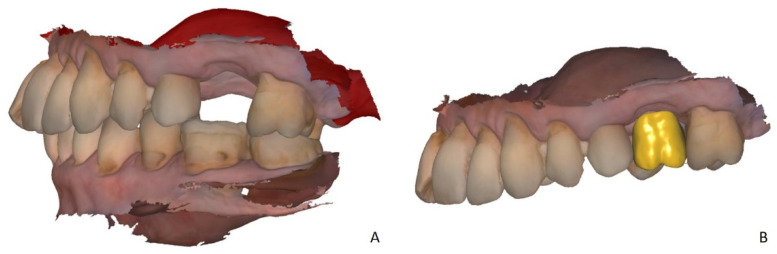
(**A**) The virtual model of the defect obtained with the intraoral scanner (Trios 4, 3Shape, Copenhagen, Denmark). (**B**) The virtual set-up of the pontic tooth in the CAD software (Dental CAD, Exocad, Darmstadt, Germany) with a 2 mm distance to the alveolar ridge.

The upper and lower jaws were scanned with an intraoral scanner (IOS) (Trios 4, 3Shape, Copenhagen, Denmark). Two side scans were performed to capture the vertical dimension of occlusion (VDO). The obtained dataset was imported in STL format in CAD Software (Dental CAD 2.3 Matera, Exocad, Darmstadt, Germany) (Figure 1A). A virtual set-up of a missing tooth was performed, leaving 2 mm space towards the alveolar ridge (Figure 1B).

**Figure 2:**
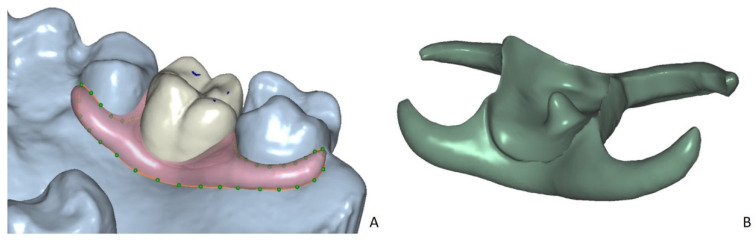
(**A**) The virtual design of the denture base in the CAD software (Dental CAD, Exocad, Darmstadt, Germany) with the applied pontic tooth, using the “attachment” function. (**B**) The final denture design with a corresponding retention socket for the pontic tooth.

After the artificial pontic tooth was adjusted in terms of shape, size, and position, its virtual design was exported in STL format. After that, a denture base was designed with the clasps on the neighbor teeth considering 2 mm thickness (Figure 2A). The pontic tooth was uploaded using the “attachment” function and then cut out from the denture base virtually, using the Boolean function in Dental CAD 2.3 (Matera, Exocad, Darmstadt, Germany) (Figure 2B).

**Figure 3:**
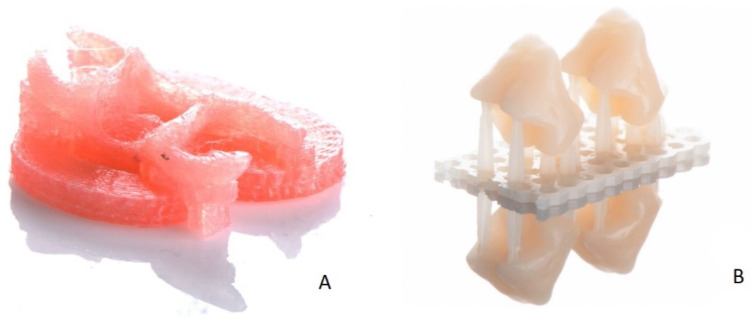
(**A**) The final denture base printed with a FFF printer (r.Pod, Arfona, NJ, USA) and polyamide filament (Thermoplastic Denture Base Resin 3D Printing Filament, Valplast International Corp, NY, USA). (**B**) The printed pontic tooth with a DLP printer (D30II, Rapidshape, Heimsheim, Germany) and tooth-colored resin (Freeprint temp A3, Detax, Ettlingen, Germany).

Both pontic tooth and denture base designs were exported in STL format. The tooth was prepared for printing in a nesting software (Netfabb Ver. 2019.2, Autodesk, Munich, Germany) and denture base in a slicing software (Simplify 3D Version 4.1, Simplify 3D, Cincinnati, Ohio, USA) to adjust the supporting structures and set the printing preferences. The denture base was printed with polyamide filament (Thermoplastic Denture Base Resin 3D Printing Filament, Valplast International Corp, NY, USA) using the FFF method (r.Pod, Arfona, NJ, USA) (Figure 3A). The melting temperature of the print head was 245 °C, and the printing table was heated to 40 °C. Afterwards, the pontic tooth was printed using the tooth-colored resin (Freeprint temp A3, Detax, Ettlingen, Germany) with a digital light processing (DLP) printer (D30II, Rapidshape, Heimsheim, Germany) (Figure 3B).

The post-processing stage considered the removal of supporting structures by both pontic tooth and denture basis. Additional post-processing was done by cleaning the printed pontic tooth in isopropanol >98% with an ultrasonic cleaner for 2 ×3 min and post-curing in a light chamber (Otoflash G171, NK Optics, Baierbrunn, Germany) under protective nitrogen for 2 ×2000 flashes. Then, the denture base surface was refined using a milling cutter. The adjacent surfaces of both pontic tooth and denture base were sandblasted with 125 µm corundum, and the pontic tooth was integrated into the base using the adhesive component (Val-Fuse, Johannes Weithas, Lütjenburg, Germany). It was further on put in the pressure pot under 55 °C and 2.5 bar for 20 min (Polymax, Dreve, Unna, Germany).

**Figure 4:**
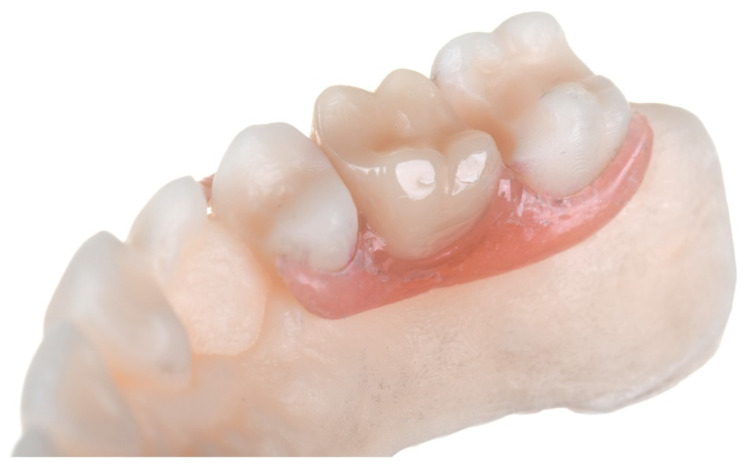
A: The finally adjusted and polished denture on the printed model.

The jaw model was printed (Freeprint model, Detax, Ettlingen, Germany) and finished (same post-process as described before) using the DLP printer (D30II, Rapidshape, Heimsheim, Germany), and the manufactured prosthesis was adjusted in terms of retention, occlusion, and approximal contacts (Figure 4). The final denture adjustment encompassed polishing in the laboratory micromotor with control box and straight handpiece, first with a polishing brush (Weiton-Smooth, Weithas) at 3000 r/min and then with polishing paste (Gold Tripoli Paste, Johannes Weithas, Lütjenburg, Germany) using a lathe bristle brush.

## 3. Discussion

The manufacturing process of a non-metal clasp RPD in a fully digital workflow has already been demonstrated in some clinical cases; however, this utilized the PMMA for the denture basis [10,13]. The present dental technique describes the direct 3D printing of a RPD using polyamide material. As the FFF method utilizes the filament fabrication process, the molten filaments can be recognized even after the layers have been merged together. This results in a poor surface quality, which necessitates efforts in post-processing, such as grinding and polishing. Even after the denture base has been polished, the inner layer-wise structure could not be entirely eliminated. Furthermore, the polyamide material is known to be susceptible to the polishing process, as it has a low melting point. Thus, polishing at a high speed may result in overheating the material, causing surface detriment and a higher surface roughness. Marchan et al. reported increased roughness (Ra) values for Valplast, compared to PMMA [20]. The study of Kraemer Fernandez et al. demonstrated that the application of coatings on the 3D-printed denture surface might result in a clinically acceptable outcome [21]. This approach might be considered for polyamide RPD in further research.

**Figure 5:**
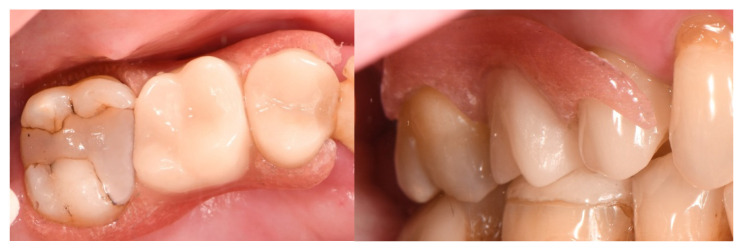
The final provisional denture in situ.

As used in the present case, the FFF printer was equipped with the 0.1 mm nozzle and provided the denture bases with acceptable clinical fit within 32 min of printing time (Figure 5). FFF is recognized as not the most precise AM method due to its relatively poor resolution in the z-axis of 0.1 mm. The selective laser sintering (SLS) was shown to cause less dimensional error (0.30%) than FFF (0.44%) [22], and the additive manufacturing of polyamide with SLS using white powder has been also demonstrated [23]. It may be considered in the future as an alternative manufacturing method of RPD once the powder color can be adjusted.

The present clinical case dealt with a single tooth defect in a tooth limited edentulous space. The replacement of a higher amount of teeth with a more extended denture base may be challenging for the FFF printing of polyamide filaments as well as the retention of such a RPD. A conventionally manufactured polyamide material was reported to have poor color stability and mechanical properties, and its utilization as an alternative to PMMA is still disputable [6,24,25]. For this reason, long-term studies are be needed to evaluate mechanical properties and clinical performance of additively manufactured PA-12-based RPDs.

## 4. Conclusions

This clinical report shows a pathway to construct a non-metal clasp RPD from polyamide material using an intraoral scanner and FFF printer. This technique allows for a sufficiently accurate and rapid fabrication of an RPD. However, regarding the mentioned limitation of polyamide material and FFF printing methods in general, the present RPD should be considered as a temporary solution in small gaps.

## Data Availability

The datasets used and/or analyzed during the current study are available from the corresponding author on reasonable request. All STL files can be transferred via cloud service on request.
